# The Role of Autophagy in Osteoclast Differentiation and Bone Resorption Function

**DOI:** 10.3390/biom10101398

**Published:** 2020-09-30

**Authors:** Azadeh Montaseri, Claudia Giampietri, Michela Rossi, Anna Riccioli, Andrea Del Fattore, Antonio Filippini

**Affiliations:** 1 Department of Anatomy, Histology, Forensic Medicine and Orthopaedics, Unit of Histology and Medical Embryology, Sapienza University of Rome, 00161 Rome, Italy; montaseri_azi@yahoo.com (A.M.); anna.riccioli@uniroma1.it (A.R.); Antonio.filippini@uniroma1.it (A.F.); 2 Department of Anatomy, Histology, Forensic Medicine and Orthopaedics, Unit of Human Anatomy, Sapienza University of Rome, 00161 Rome, Italy; claudia.giampietri@uniroma1.it; 3 Bone Physiopathology Research Unit, Genetics and Rare Diseases Research Division, Bambino Gesù Children’s Hospital, IRCCS, 00165 Rome, Italy; michela_r10@yahoo.it

**Keywords:** autophagy, osteoclast, differentiation

## Abstract

Autophagy is an evolutionary conserved and highly regulated recycling process of cellular wastes. Having a housekeeping role, autophagy through the digestion of domestic cytosolic organelles, proteins, macromolecules, and pathogens, eliminates unnecessary materials and provides nutrients and energy for cell survival and maintenance. The critical role of autophagy and autophagy-related proteins in osteoclast differentiation, bone resorption, and maintenance of bone homeostasis has previously been reported. Increasing evidence reveals that autophagy dysregulation leads to alteration of osteoclast function and enhanced bone loss, which is associated with the onset and progression of osteoporosis. In this review, we briefly consolidate the current state-of-the-art technology regarding the role of autophagy in osteoclast function in both physiologic and pathologic conditions to have a more general view on this issue.

## 1. Bone Remodeling

Bone is a specialized type of connective tissue that holds several important functions including mechanical support of vital organs, locomotion, storage of mineral ions, hormone secretion, modulation of cognition, and anxiety-like behavior that provides the niche site for bone marrow. To maintain its integrity and function, bone is subjected to a continuous process of remodeling by which the old matrix is eliminated through activation of osteoclasts and new formed matrix is replaced by osteoblasts [1]. Bone remodeling consists of three consequent phases. The first one is the initiation phase in which bone matrix is resorbed through osteoclast activity. The second step is called transition/reserve phase and is the gap between the end of resorption step and the beginning of bone formation. In this phase, the so-called reverse cells appear, whose role has not yet been completely clarified. They are macrophage-like cells with a likely function of removal of debris produced during matrix degradation. The third phase is the bone formation [2,3,4].

Pre-osteoclasts originate from hematopoietic myelomonocytic cells. They are attracted to the bone resorption sites and fuse to form terminally-differentiated multinucleated osteoclasts [5,6].

Osteoclasts are giant cells that resorb bone. Upon attachment to the bone matrix surface by highly dynamic structures called podosomes, osteoclasts become polarized and form a “ruffled border.” This is the bone-facing domain characterized by deep and irregular folding of the membrane. Across the ruffled border, protons and lysosomal degrading enzymes are transferred to the underlying bone to erode the bone matrix [7]. The secretion of protons by Vacuolar (V)-H+ATPase and Cl- by the chloride proton antiporter CLC-7 lowers the pH in the resorption lacuna to about 4.5, which leads to the demineralization of the bone matrix and unmasking the organic components mainly composed of type 1 collagen [8,9,10]. In the next step, exposed collagen and other organic components are degraded by lysosomal enzymes such as cathepsin K and MMPs (matrix metalloproteinases) released by the fusion of lysosomes with the ruffled border [11,12,13].

The bone matrix resorption leads to the release of molecules embedded in the bone matrix as TGF-β (transforming growth factor β), which hires pre-osteoblasts to the bone [7]. Furthermore, osteoclasts release HGF (hepatocyte growth factor), S1P (sphingosine-1-phosphate), and TRAP (tartrate-resistant acid phosphatase) to promote the proliferation and motility of osteoblasts [7,14]. Osteoblasts express high levels of ALP (alkaline phosphatase) and generate unmineralized and flexible bone osteoid by secreting matrix proteins including type I collagen (90%) and a small percentage of proteoglycans, fibronectin, and specific bone proteins, such as osteopontin and osteocalcin [15]. Subsequently, osteoblasts release matrix vesicles or calcifying globules by budding, which leads to the formation of hydroxyapatite crystals made of calcium and phosphate [Ca_10_(PO_4_)_6_(OH)_2_] [7].

At the end of the bone formation phase, osteoblasts may be subjected to apoptosis, become bone lining cells, or are trapped in the bone matrix as osteocytes, which are non-proliferative terminally differentiated cells. Osteocytes are fusiform, flat-shaped cells with long dendritic processes rich in actin filaments, which speed up and integrate the communication between osteocyte network and also with the bone surface [16,17,18,19].

Osteoblasts and osteocytes can regulate osteoclastogenesis since they release M-CSF (macrophage colony stimulating factor), which binds to its receptor c-Fms located on the osteoclast progenitors and induces their proliferation. Moreover, they release RANKL (Receptor activator of nuclear factor-kappa B ligand) that binds to its receptor RANK expressed on osteoclast precursors and osteoclasts, which regulate the differentiation, survival, and activity of mature osteoclasts [2,7]. Binding of RANKL to RANK receptor results in the attachment of TRAF6 [TNFR (Tumor necrosis factor receptor)-associated cytoplasmic factor-6) to the intracellular domain of RANK [1,20,21]. TRAF6 activates MAPKs (mitogen-activated protein kinases), ERK (extracellular signal-regulated kinase), JNK (c-Jun N-terminal kinase), p38 signaling, and NF-kB (nuclear factor-kappa B) to regulate the expression of osteoclast factors. One of the early key target genes of NF-kB is NFATc1 (nuclear factor of activated T cells calcineurin-dependent 1) that is also one of the downstream targets of RANK. Activation of NFATc1, in turn, promotes the expression of osteoclast specific genes encoding TRAP, DC-STAMP (dendritic cell specific transmembrane protein), V-H+ATPase, and OC-STAMP (calcitonin receptor and osteoclast stimulatory transmembrane protein) [6,22,23].

Osteoblasts and osteocytes produce OPG (osteoprotegerin), a decoy receptor for RANKL, which binds to the cytokine and prevents its interaction with RANK, interrupting osteoclastogenesis [24].

Under normal conditions, there is a sharp equilibrium between bone formation and resorption and its disruption can lead to pathological conditions [25]. This dynamic balance can be influenced by hormones, growth factors, and mechanical impulses as extrinsic factors and autophagy as an intrinsic one [16,25,26].

## 2. Autophagy and Bone Remodeling

Maintenance of cellular homeostasis depends on a precise balance between synthesis and destruction of organelles and macromolecules [27,28], which, in eukaryotes, is regulated by two degradative processes including autophagy and proteasomes [28]. The term “autophagy” comes from two Greek roots in which “autos” means self and “phagein” means eat. This term for the first time was coined by Christian de Duve in 1963 [29].

Autophagy is a highly conserved catabolic process in eukaryotic cells in which cytoplasmic materials such as macromolecules, organelles, and exogenous pathogens are sequestered and degraded [30]. During this cell cleaning mechanism, targeted organelles and materials are engulfed within a double-membraned vesicle called autophagosome. By fusion to the lysosome and formation of autolysosome, the content of autophagosome is degraded and recycled, which provides raw nutrients and energy for cell repair and survival [25,31]. In healthy cells and under physiological conditions, autophagy occurs at a low level and serves as a quality-control system for maintaining cell homeostasis [32,33] and has a crucial role particularly in highly-differentiated cells like myocytes, osteocytes, and neurons [34]. Under different stress conditions, such as nutrient and energy starvation, hypoxia, and infection, the rate of autophagy increases to promote recycling of cytoplasmic components and cell survival [26,34,35,36,37].

In mammals, there are three types of autophagy, which differ for the type of cargo, regulatory mechanisms, and initiating signals: chaperone-mediated autophagy, micro-autophagy, and macro-autophagy [25,28,38,39].

In chaperone-mediated autophagy, the cargo is delivered to the lysosome by cytosolic chaperones such as heat shock proteins. Inside the lysosomal lumen, these macromolecules are unfolded and then exit separately throughout the lysosome membrane [40]. In micro-autophagy, the cargo enters into the lysosome by invagination on its membrane [41]. In macro-autophagy (hereafter referred as autophagy), the cargo is captured and engulfed in a double-membraned vesicle named as autophagosome, which fuses to lysosome and forms autolysosome for further digestion and recycling of its contents [25].

Autophagy is regulated by a family of highly conserved genes called autophagy-related genes (ATG), which are approximately 20 members.

The autophagy process can be divided into four stages: initiation/nucleation, elongation, degradation, and termination [42]. In response to insufficiency of cellular nutrients, the initiation phase of autophagy starts by activation of the ULK-1 (Unc-51-like kinase 1) complex, which is composed of ULK-1, Atg13, Atg101, and focal adhesion kinase family-interacting protein named FIP200 [41]. Before starting the autophagy, the ULK-1 complex is in association with mTORC1. By initiation of autophagy, the ULK-1 complex is dephosphorylated, activated, and separated from mTORC1. This complex subsequently activates the VSP34 complex (VSP34, Beclin-1, VSP15, and ATG14) to promote PI3P (phosphatidylinositol 3-phosphate) synthesis and nucleate cup-shaped, double-membraned phagophore. Among these proteins, Beclin-1 plays an important role for the phagophore formation and it is used as an autophagy marker [43]. In the next step, phagophore elongates to form the autophagosome. Phagophore elongation is mediated by a group of Atg proteins belonging to two Ubiquitin-like conjugation systems including Atg12 and Atg8. Atg12 is coupled with Atg5 to form an Atg12–Atg5 conjugate, whereas Atg8, often referred to as LC3 (microtubule associated protein light chain 3), is conjugated to a lipid, phosphatidylethanolamine to form LC3II, which integrates to the phagophore membrane and assists its elongation and closure [42,44]. While phagophore expands, it engulfs the cargo and forms autophagosome. In the degradation step, autophagosome merges with endocytic compartments (early and late endosomes) to form amphisome [27,45], which later fuses with the lysosome to establish an autolysosome. This phase allows the acidification and degradation of the delivered macromolecules into amino acids, lipids, nucleotides, and energy for further use by the cell [46]. Several proteins are involved like dynein, which regulates vesicular transport, Rab7, Beclin-1, SNAREs (soluble N-ethylmaleimide-sensitive fusion protein attachment protein receptors), and many others that facilitate autophagosome maturation [24,25]. Some proteins such as p62, NBR1 (neighbor of BRCA1 gene), and ALFY (autophagy-linked FYVE protein) are located at the autophagosome surface and sequester the degradation targets [25]. The p62 protein, also known as SQSTM1 (sequestosome 1), is involved in selective autophagy by binding to LC3II on the surface of the autophagosome and to ubiquitinated proteins on the other side by delivering them inside the autophagosome. During this mechanism, p62 enters the autophagosome and degrades. Since p62 is considered a marker for the autophagosome, change of its expression level is used to understand the decline or augmentation of the autophagy process [47,48]. The last step in the autophagy process is termination. During this step, mTOR reactivates and forms tubules and vesicles from the autolysosome, which later merge together and generate new lysosomes [49]. In general, as a sharply regulated intracellular degradation and recycling process, autophagy is associated with the maintenance of the cellular homeostasis and survival.

Having a broad contribution in most biological processes, it is not surprising that impaired autophagy can play an important role in the onset and development of several pathological conditions including neurodegenerative diseases, cancer, and heart disease [50,51]. Furthermore, a great body of evidence showed the role of autophagy in the differentiation and activity of different bone cells and its dysfunction is associated with many bone-related disorders including osteoporosis, osteopenia, and Paget’s disease [30,52]. The role of some autophagic proteins on bone cells was demonstrated by studying genetic animal models as reported in Table 1.

A basal level of autophagy is required to maintain the stemness, replicative, and differentiation capacity of stem cells [59,60], protect bone marrow macrophages from oxidative stress [61], and MSCs (mesenchymal stem cells) against cell aging [59,62,63,64,65].

A great body of evidence revealed that increased autophagy occurs during osteoblast differentiation and mineralization [66,67]. Mineral formation by osteoblasts is proposed to be initiated inside vesicles, either after their secretion, as in matrix vesicles, or before their secretion. Inside the cell, the mineralization can occur in autophagy-like vesicles. These vesicles assume as cargos for transporting mineral needle-like crystals containing calcium and phosphate, which can facilitate the bone matrix mineralization. Moreover, the inhibition of the autophagy pathway results in impairment of bone mineralization and decrease of bone mass in an in vivo condition [67]. However, the role of autophagy in other calcification processes is different. It was demonstrated that the inhibition of autophagy in vascular smooth muscle cells increases the vascular calcification by protecting the cells from apoptosis and stimulating the release of vesicles with increased ALP activity [68].

In bone, autophagy ablation leads to the osteoblast dysfunction through endoplasmic reticulum (ER) stress and also increases the secretion of RANKL, which results in the activation of osteoclasts and bone resorption [26]. Autophagy also regulates osteocytes. When compared to the osteoblasts, osteocytes live in the mineralized matrix with poor blood perfusion and are more susceptible to hypoxia and a high level of oxidative stress. Living in such a situation, osteocytes show an elevated level of autophagy and higher number of autophagosomes compared to osteoblasts [69,70,71]. Additionally, pharmacological induction/inhibition of autophagy leads to alterations of the osteocyte apoptosis rate [72,73]. This information suggests the indispensable role of autophagy in maintaining osteocyte function and keeping the balance of bone remodeling.

The increased level of autophagic proteins during osteoclast differentiation has been reported [5,74,75]. Furthermore, it has been revealed that autophagic-related proteins are crucial in osteoclast activity since they regulate ruffled border formation, lysosomal trafficking, and secretion in both in vitro and in vivo conditions [53].

## 3. Autophagy in Osteoclast Differentiation/Function

### 3.1. Regulation of Osteoclast Differentiation by Autophagy

Living at the surface and interior parts of the bone, osteoclasts encounter a low oxygen tension in their local environment [16]. Different studies have reported that hypoxia via activation of HIF-1α (hypoxia inducing factor-1α) enhances the osteoclast differentiation and activity along with autophagic flux [76]. HIF-1α induces the expression of its downstream target BNIP3 (Bcl-2/adenovirus E1B 19 kDa interacting protein 3), which stimulates Beclin-1 release, increases the expression level of autophagic-related genes such as Atg5 and Atg12, recruits LC3 to autophagosome, and enhances the expression of RANKL, cathepsin K, NFATc1, and MMPs, which leads to increased osteoclastogenesis [77].

Sun et al. characterized the regulatory axis of HIF-1α–miRNA-20a–Atg16l1 in hypoxia-induced osteoclast differentiation. In this study, miRNA20 was identified as the candidate miRNA target for Atg16l1 gene that is involved in LC3 lipidation for autophagosome formation. Under hypoxic conditions, miRna20a significantly reduced the expression of NFATc1, TRAF6, and TRAP, and the number of TRAP positive cells [78]. The relevance of miRNAs in interplay between autophagy and osteoclastogenesis was revealed in a lipopolysaccharide-induced inflammatory condition. It was shown that miR-155 directly induces autophagy in osteoclasts by regulating their differentiation and activity by targeting TAB2 (TGF-β-activated kinase 1-binding protein 2) [79].

The tight association between autophagy and osteoclastogenesis was further demonstrated by drug treatments that regulate the two processes. It was demonstrated that 3-methyladenine inhibits autophagy and osteoclastogenesis, and that 1a,25-(OH)2 Vitamin D3 induces the autophagy of osteoclast precursors both in the absence and presence of RANKL, which reduces their proliferation and promotes osteoclast differentiation [80].

Lin et al. (2016) demonstrated that selective deletion of Atg7 in osteoclast precursors of mice ameliorated the bone loss and osteoclast hyperactivation induced by glucocorticoid or ovariectomy [57]. They also showed that pharmacological inhibition of autophagy diminished the expression of NFATc1, TRAP, and cathepsin K in osteoclasts. GIT1 (G-protein coupled receptor kinase 2 interacting protein 1) is a scaffold protein that interacts with several signaling molecules [81,82], and also mediates osteoclast differentiation, bone mass regulation, and fracture healing [82,83]. The work of Zhao et al. extended these findings and showed reduction of starvation-induced autophagy due to GIT1 knockdown and promotion of autophagic flux due to GIT1 overexpression. Furthermore, GIT1 stimulates the phosphorylation of Beclin-1 and dissociation of Beclin-1 from Bcl-2 (B-cell lymphoma 2) that positively regulates autophagy process. Knockdown of GIT1 also decreased the number of osteoclasts at the site of bone fracture in mice [81].

p62 plays an important role in osteoclastogenesis, as shown when studying Paget’s disease of bone characterized by focal areas of abnormal, excessive bone turnover, which specifically increased bone resorption and disorganized bone formation. p62/SQSTM1 is a key regulator of ubiquitinated protein turnover through autophagy and ubiquitin-proteasome system. Until recently, the role of p62 in osteoclasts was unclear, but there is strong evidence to suggest that p62 recruits the deubiquitinating enzyme CYLD (Cylindromatosis) to RANK-TRAF6 complex. In this way, CYLD inhibits RANK signaling by deubiquitinating TRAF6. In patients affected by Paget’s disease with mutations of SQSTM1, defective p62 does not allow the CYLD protein to bind to the TRAF6 complex by causing osteoclast differentiation and activation [84].

Arai et al. demonstrated that TRAF-mediated Beclin-1 ubiquitination is necessary for RANKL-stimulated osteoclast differentiation, and Beclin-1 knockdown in mice led to the thickening of the cortical bone due to impaired bone resorption [57]. One of the main signaling pathways involved in the RANKL/RANK system is Ca^2+^-calcineurin-NFATc1 [85]. The influx of Ca^2+^ is essential for osteoclastogenesis and it is mediated by a Ca^2+^-permeable channel named TRPV4 (Transient receptor potential vanilloid 4) [86]. In a study by Cao et al., it was reported that 3-methyladenine and autophagy suppression attenuated the TRPV4-induced osteoclastogenesis and TRPV4 knockdown significantly restored BMD (bone mineral density) in ovariectomized mice [87].

### 3.2. Regulation of Osteoclast Migration by Autophagy

The migration of osteoclasts on the bone matrix is essential for their bone resorption activity. This process is regulated by rapid and successive assembly and disassembly of podosomes. Podosomes are organized by the dot-like core of actin filaments surrounded by a ring-like of actin regulatory proteins such as integrin-associated proteins (vinculin and kindlin3) and kinases (PI3K and Rho GTPases) [88].

In a recently published paper, Zhang et al. demonstrated that LC3B acts in the disassembly of podosomes and regulates osteoclast migration by the adaptator protein kindlin3. A downregulation of LC3B enhanced the interaction between integrin β3 and kindlin3. LC3B-deficient osteoclasts fail to degrade old podosomes by increasing the number of podosomes and ring thickness, which leads to podosome ring dysfunction and affects osteoclast ability to resorb bone [89].

### 3.3. Regulation of Bone Resorption by Autophagy

**Figure 1:**
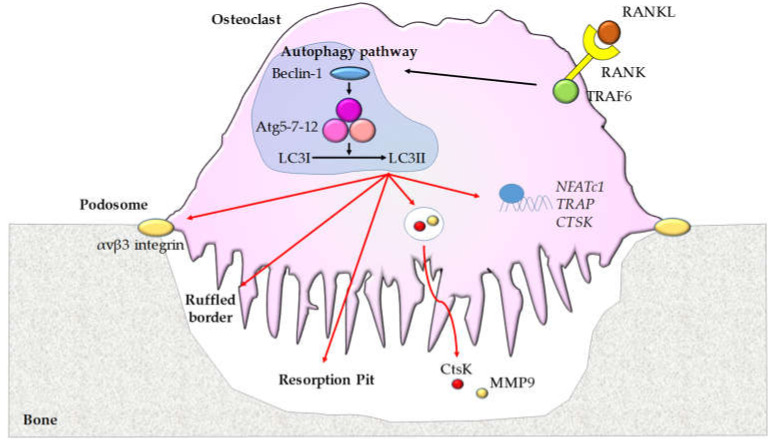
The role of autophagy in osteoclasts. Osteoblast-secreted and osteocyte-secreted RANKL that binds to its receptor RANK on osteoclasts leads to the recruitment of TRAF6 and an increase of Beclin-1 and Atg5/7/12 with enhanced conversion from LC3I to LC3II. This autophagic pathway regulates the expression of osteoclast genes [*NFATc1*, *TRAP*, and *CTSK* (Cathepsin K)], podosome and ruffled border formation, and bone resorption activity stimulating the secretion of CtsK and MMP9.

In addition to the role in osteoclast differentiation, autophagy also regulates the bone resorption activity (Figure 1).

Delivery of Bafilomycin A1, which is a specific inhibitor of V-H+ATPase activity and autophagy, to the tooth eruption site results in the blocking of bone resorption and inhibition of tooth eruption [90]. Moreover, De Selm et al. showed that lysosomal fusion to the ruffled border and secretion of their contents are mediated by autophagy proteins. They also reported that no autophagosome was observed near the ruffled border, which suggests that autophagosomes do not contribute to the ruffled border but autophagy-dependent molecules participate in the delivery of secretory lysosomes to this membrane. Impaired activation of these autophagy proteins leads to the defective bone resorption due to a lack of a ruffled border, disrupted localization of cathepsin K and LAMP1 within the actin ring, and impaired fusion of lysosomes to the plasma membrane. Specific knockdown of Atg5 or Atg7 in osteoclasts inhibited LC3I to LC3II conversion and decreased the resorption activity, but did not suppress osteoclast differentiation, actin ring formation, and lysosome biogenesis. Moreover, when Atg5 was knocked down, significant downregulation in Cdc42 (Cell division cycle 42) activity and actin-ring disruption were observed [58]. Its deletion reduced ovariectomy-induced bone loss in mice, which suggests the role of autophagy proteins in regulation of osteoclast function in normal and pathological conditions [53]. Beyond playing a role as an autophagy protein, LC3 also acts as a mediator in controlling the dynamic microtubule network in osteoclasts. Knockdown of LC3 suppresses actin ring formation, cathepsin K release, and bone resorption activity but not the number of TRAP positive cells. Chung et al. identified that LC3 mediates these effects via association with Cdc42, which is a key regulator of the actin cytoskeleton [58].

Autophagy plays dual roles in the regulation of osteoclasts. Even if autophagy is important for the bone resorption, it does not mean that its activation necessarily stimulates osteoclast activity [91]. OPG inhibits osteoclast differentiation and activity, and also influences the autophagy pathway upregulating the expression of Atg5, 7, 12, 13, Beclin-1, and LC3I/II ratio in bone marrow macrophages [92]. OPG activates the AMPK protein, which leads to inactivation of mTORC1 whose inhibition enhances autophagy [93].

The relevance of mTOR was further demonstrated. The induction of autophagy by mTOR inhibitor rapamycin in young rats decreased the RANKL expression, the number of TRAP-positive multinucleated cells, and the endochondral bone growth [94]. Furthermore, inhibition of mTOR by rapamycin down-regulated the expression of degrading enzymes and induced osteoclast apoptosis [95]. Renal transplanted patients treated with rapamycin are characterized by a reduced serum level of bone resorption markers due to inhibition of osteoclast differentiation and resorption activity [96].

## 4. The Role of Autophagy in Bone Disorders

The tightly coupled function of osteoblasts and osteoclasts is fundamental to maintain the normal bone density to repair the microcracks in the bone matrix to avoid accumulation of the old bone matrix and to regulate mineral homeostasis [97,98]. Its alteration leads to bone disorders like osteoporosis [98,99]. Osteoporosis, which is a socioeconomic global problem, is defined in terms of BMD and fracture as a T-score [number of standard deviations that a measured BMD differs from the mean BMD of a young adult population (T-score)] of ≤ −2.5 and/or a previous fragility fracture [25,100]. The most prevalent form of osteoporosis is the postmenopausal one, which occurs as a consequence of sex steroid loss [101]. Estrogens play an important role in controlling the function of bone cells and maintaining the bone density.

An association between estrogen and autophagy-related proteins in different organs has been reported [36,102,103,104]. In a study performed as early as the 1970s, the relationship between castration and increased level of autophagy and lysosome number was stated [105]. Since autophagy is involved in the activity of all bone cells, its disturbance is associated with several bone disorders as osteoporosis [25]. It was demonstrated that autophagy activation in osteoclasts is responsible for the rapid bone loss in ovariectomized mice [53]. 

Xiu et al. demonstrated that pharmacological inhibition of autophagy by chloroquine reduced the osteoclast number and surface preventing ovariectomy-induced bone loss [106]. The protective effect of the drug was also demonstrated for the bone loss induced by glucocorticoid since it suppressed osteoclastogenesis [53]. To confirm the role of autophagy in the bone erosion, it was demonstrated that the selective deletion of Atg7 in monocytes mitigated the osteoclast differentiation and bone loss induced by glucocorticoid and ovariectomy [53]. Similarly, the selective deletion of Atg5 in osteoclasts decreases about 50% the bone loss induced by estrogen deficiency and reduced the level of the bone resorption marker CTX (C-terminal telopeptide of type I collagen) [53].

The aging also affects the skeleton since it reduces the bone mass and induces changes in matrix composition, which leads to fragility and increased risk of fracture [107].

**Figure 2:**
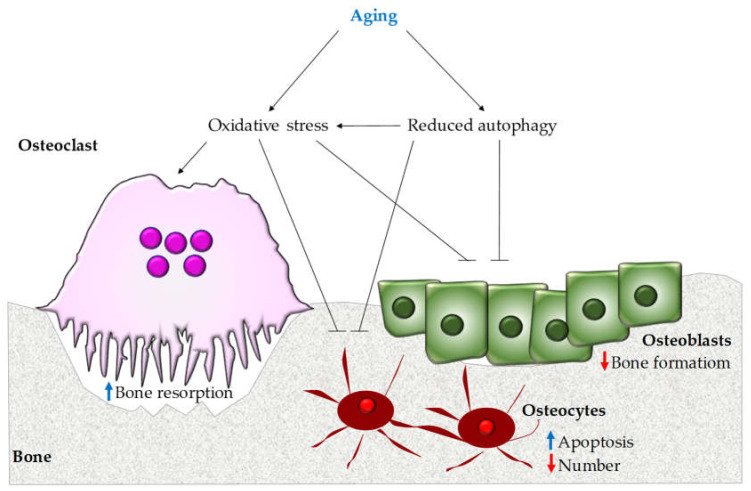
Aging-associated alteration of autophagy. Aging triggers the onset of osteoporosis since it reduces autophagy, which leads to inhibition of bone formation by osteoblasts and induces the apoptosis of osteocytes. This results in increased oxidative stress that stimulates bone resorption and osteocyte apoptosis and decreases osteoblast activity.

The aging down-regulates the level of macro-autophagy particularly in terminally differentiated cells like osteocytes concurring to age-related diseases [108,109]. Since autophagy has antioxidant effects, its decline by aging results in increased oxidative stress in the bone microenvironment, which contributes to the bone loss (Figure 2) [110].

In addition, loss of estrogen is associated with a decreased defense of bone cells against oxidative stress and leads to bone resorption and decreased bone mass in menopausal women [72,111]. A high levels of AOPPs (advanced oxidation protein product), which is a novel biomarker of oxidative stress, have been reported in postmenopausal osteoporotic women and are correlated with reduced bone mass density [112]. Elevated systemic inflammation, which is commonly seen in elders (named inflamm-aging) is involved in age-related morbidities [113,114]. Persistent activation of innate inflammatory signaling has a deleterious effect on bone cell activity that involves the NF-kB pathway [25]. Its activation in differentiated osteoblasts shows anti-anabolic effects and impairs bone formation in osteoporosis [115]. Moreover, NF-kB stimulates osteoclast differentiation and mediates bone resorption [116], which demonstrates the involvement of constitutive activation of the inflammatory pathway in age-related osteoporosis. In addition, autophagy-associated proteins are involved in inflammatory signaling in both autophagy dependent and independent manners. In autophagy dependent way, Atg proteins decrease the level of ROS and RIG-I (retinoic acid-inducible gene-1) and remove the damaged mitochondria, while, in the autophagy independent process, Atg proteins interact directly with inflammatory molecules [25]. For example, the Atg5-Atg12 conjugate has a pivotal function in the innate antiviral immune responses by suppressing immunostimulatory RNA and Interferon type I production [117]. Correspondingly, Beclin-1 stimulates autophagy-related pathogenic DNA degradation and blocks Interferon production by suppressing cyclic GMP-AMP synthesis [118]. Taken together, these data highlight the role of autophagy and autophagy-related proteins in controlling the inflammatory pathway. The increase of systemic inflammation and the reduction of the autophagic capacity limit the ability of bone cells to defend themselves against oxidative stress and lead to the impairment of bone homeostasis by aging. This reduced autophagy capacity, which occurs by aging and estrogen deficiency in post-menopausal women, promotes bone loss and osteoporosis.

Autophagy pathways are also involved in the bone loss associated with microgravity [119]. It has been reported that astronauts, who experienced long-duration space missions in microgravity, lose bone mass and encountered fracture risk [120,121]. Mononuclear precursor cells cultured in the perfusion bioreactor system mimicking microgravity showed a severe increase of TRAP positive cells and expression level of osteoclast-specific genes [122,123]. Sambandam et al. evaluated the involvement of autophagy in weightlessness-induced osteoclastogenesis and reported up-regulation of autophagy-associated molecules such as Trp53 (Transformation-related protein 53), Prkaa1 (Protein kinase AMP-activated catalytic subunit alpha 1), autophagosome components, and markers such as Atg5, 16L, 9b, and LC3. Furthermore, 3-methyladenine treatment of mouse bone marrow non-adherent cells exposed to microgravity resulted in significant down-regulation of autophagosome markers expression and osteoclast differentiation [119].

The involvement of autophagy in the pathogenesis of rheumatoid arthritis was also demonstrated. TNF (Tumor Necrosis Factor), which plays a crucial role in the pathogenesis of the disease, regulates osteoclast differentiation and activation via stimulation of the autophagy pathway [124]. Moreover, the expression level of Atg7 and Beclin-1 in osteoclasts isolated from patients is higher than in healthy donor cells [125]. Lin and colleagues reported that TNF stimulates the LC3I conversion into LC3II and induces the expression of Atg7 and Beclin-1 in murine osteoclasts [124].

TNF stimulates osteoclast precursor differentiation via TRAFs and NF-kB [126], but it can also reduce osteoclastogenesis through stimulation of NF-kBp100 and TRAF3 levels [127]. Xiu et al. demonstrated that RANKL can degrade TRAF3 by activating autophagy and chloroquine as an autophagy inhibitor reduces RANKL-mediated osteoclastogenesis and increases TRAF3 expression in bone marrow macrophages [106].

MCP-1 (Monocyte chemotactic protein-1) is a CC chemokine, expressed by mature osteoclasts, which is found at the place of bone loss, tooth eruption, and degraded bone in patients affected by rheumatoid arthritis [128]. MCP-1 is considered to be induced by NF-kB and enhances osteoclast precursors’ fusion [129,130]. Activation of RANK stimulates MCP-1 binding to CCR2, a G-protein-coupled receptor, which further leads to activation of a novel zinc-finger protein, MCPIP (MCP-induced protein), in human monocytes [131]. MCPIP induces ROS formation and endoplasmic reticulum stress that causes up-regulation of Beclin-1, lipidation of LC3, and expression of TRAP and cathepsin K [132]. These effects are sequestered by treatment of monocytes with 3-methyladenine and Beclin-1 or Atg7 knockdown, which underlines that osteoclast differentiation by MCPIP is mediated through ROS synthesis and ER stress by leading to autophagy [132].

These results suggest that factors and agents with the ability to modulate the autophagy process can be introduced as potential therapeutic candidates for bone diseases. Moreover, it has long been recognized that the autophagy level should be maintained in a narrow range of homeostasis and the balance between the autophagy process and other cellular activities is so delicate that alterations may lead to cellular death. At the same time, it is of great importance to notice that different compounds, which are able to augment autophagy, exert several systemic side effects on patients [133]. Therefore, development of cell or organ-specific autophagy modulators could be a possible solution for patients suffering from osteoporosis. 

## 5. Conclusions

In summary, autophagy as a well conserved self-eating and self-editing mechanism has a critical role in maintaining the normal formation and activation of bone cells other than osteoclasts and is fundamental for keeping the physiological bone homeostasis. It is not surprising that its malfunction and dysregulation influence the bone health. By age advancing, the increased level of ROS, loss of estrogens, and decreased autophagic capacity, lead to the limited cell capacity to defend itself against intracellular and extracellular stimuli. As a result of autophagy deficiency and increased oxidative stress, osteoclasts behave in a pathologic manner that results in excess bone loss and osteoporosis. Thus, this fundamental and complicated role of autophagy in controlling the bone density and remodeling suggests autophagy-related proteins as novel targets for future therapies of skeletal diseases. Furthermore, it is of great importance to underline that, despite the huge amounts of studies in this field in recent decades, there are plenty of unsolved questions regarding this issue and further investigations are necessary to develop new therapeutic approaches.

## Figures and Tables

**Table 1:** Knockdown of autophagy genes and effects on bone remodeling activity.

Knock Downed Gene	Effect on Bone Cells
*ATG5*	Reduction of osteoblast and osteoclast differentiation [53]
*ATG7*	Reduction of osteoblast differentiation [54] Impaired cathepsin K secretion by osteoclast and impaired bone resorption [55] Osteocyte-mediated induction of aging-like phenotype [54,56]
*Beclin*	Reduction of mineralization activity by osteoblasts [57]
*LC3*	Impairment of bone resorption [58]
